# Proprotein Convertase Subtilisin/Kexin Type 9 and Atherosclerotic Plaque Progression: A Systematic Review and Meta-Analysis of Intravascular Imaging Studies

**DOI:** 10.31083/RCM46547

**Published:** 2026-03-10

**Authors:** Panagiotis Theofilis, Panayotis K. Vlachakis, Paschalis Karakasis, Panagiotis Iliakis, Konstantinos Pamporis, Evangelos Oikonomou, Kyriakos Dimitriadis, Konstantinos Tsioufis, Dimitris Tousoulis

**Affiliations:** ^1^Cardiology Department, “Hippokration” General Hospital, University of Athens Medical School, 11527 Athens, Greece; ^2^Cardiology Department, “Hippokration” General Hospital, Aristotle University of Thessaloniki, 54642 Thessaloniki, Greece; ^3^Cardiology Department, “Sotiria” Regional Hospital for Chest Diseases, University of Athens Medical School, 11527 Athens, Greece

**Keywords:** PCSK9 inhibitor, atherosclerosis, coronary artery disease, atheroma volume, lipid arc, fibrous cap thickness

## Abstract

**Background:**

Proprotein convertase subtilisin/kexin type 9 inhibitors (PCSK9is) have emerged as a promising class of medications, primarily recognized for inducing potent cholesterol-lowering effects. In addition to the established role of these inhibitors in reducing low-density lipoprotein cholesterol levels, recent studies suggest that PCSK9is may also modify coronary atherosclerotic plaques. Therefore, this meta-analysis aimed to comprehensively synthesize data from relevant clinical studies and trials investigating the effects of PCSK9is on coronary atherosclerotic plaque characteristics.

**Methods:**

We performed a literature search for studies assessing the evolution of coronary atherosclerotic plaques after treatment with a PCSK9i compared with a control group. We excluded reviews, editorials, case reports/case series, and studies that did not use PCSK9is or lacked a control group. The main outcomes of interest were changes in percent atheroma volume (PAV), total atheroma volume (TAV), minimal fibrous cap thickness (FCT), lipid arc, and the number of patients with improved PAVs at follow-up. Effect sizes are presented as a standardized mean difference (SMD) or risk ratio (RR) alongside the corresponding 95% confidence intervals (CIs) and were pooled based on a random-effects model. Publication bias was assessed by funnel plot inspection and Egger's regression test.

**Results:**

The literature search yielded 142 results. After applying the exclusion criteria, nine studies were selected for data extraction and inclusion in the meta-analysis. Concerning the intravascular ultrasound findings, PCSK9is significantly reduced the TAV (MD –7.09 mm^3^, 95% CI –11.36 to –2.81; *p* = 0.01) and induced non-significant reductions in the PAV (MD –1.91%, 95% CI –5.08 to 1.25; *p* = 0.17); meanwhile, a greater proportion of patients treated with PCSK9 inhibitors exhibited an improvement in the PAV (RR 1.30, 95% CI 1.19 to 1.42; *p* < 0.001). For optical coherence tomography parameters, patients treated with PCSK9 inhibitors showed an increase in minimal FCT (MD 36.25 μm, 95% CI 0.75 to 71.75; *p* = 0.047) and a non-significant decrease in lipid arc (MD –17.64°, 95% CI –49.73 to 14.44; *p* = 0.14).

**Conclusions:**

This meta-analysis suggests that PCSK9i therapy may be associated with modest favorable changes in selected intravascular imaging markers of coronary atherosclerotic plaque burden and stability.

## 1. Introduction

Coronary artery disease (CAD) is driven by the accumulation and destabilization of atherosclerotic plaque, which underlies most acute coronary syndromes [1]. While statins remain the cornerstone of lipid-lowering therapy and have been shown to slow plaque progression and promote regression, residual cardiovascular risk persists. This has fueled interest in additional lipid-lowering strategies that target low-density lipoprotein cholesterol (LDL-C) more aggressively and potentially influence plaque characteristics beyond traditional statin therapy [2, 3].

Proprotein convertase subtilisin/kexin type 9 inhibitors (PCSK9is) have emerged as potent LDL-C–lowering agents, consistently reducing cardiovascular events in recent studies [4, 5]. Beyond lipid reduction, their impact on atherosclerotic plaque morphology and stability has been investigated using intravascular imaging modalities such as intravascular ultrasound (IVUS), optical coherence tomography (OCT), and near-infrared spectroscopy (NIRS). These techniques allow detailed assessment of plaque burden, composition, and vulnerability features [6], providing mechanistic insights into how PCSK9is may translate into improved clinical outcomes. Specifically, IVUS is able to characterize plaque burden through the measurement of percent and total atheroma volume (PAV, TAV), while OCT, with its superior axial resolution, can accurately estimate fibrous cap thickness (FCT) and lipid arc [7, 8]. However, the overall effect of PCSK9is on plaque progression and stabilization is not fully established. The aim of this meta-analysis is to systematically evaluate and quantify the effects of PCSK9is on atherosclerotic plaque progression and composition as assessed by intravascular imaging studies.

## 2. Materials and Methods

### 2.1 Search Strategy, Study Selection, and Outcomes

We systematically searched PubMed, Scopus, and Web of Science databases for studies published in English from inception to 30 June 2024. The full search query was the following: (PCSK9 OR alirocumab OR evolocumab OR inclisiran OR “proprotein convertase subtilisin/kexin type 9”) AND (“percent atheroma volume” OR “total atheroma volume” OR “fibrous cap thickness” OR “lipid arc”).

We included randomized controlled trials, non-randomized trials, and cohort studies that assessed the effect of PCSK9is on coronary atherosclerotic plaques using intravascular imaging. Eligible studies enrolled adult patients with coronary artery disease who received treatment with a PCSK9i (evolocumab, alirocumab, or inclisiran) compared with either placebo, standard lipid-lowering therapy, or no PCSK9i therapy. The primary outcomes of interest were changes in PAV and TAV. Secondary outcomes included changes in minimal FCT, lipid arc, and the proportion of patients demonstrating PAV regression at follow-up.

Studies were considered suitable if they reported at least one of the following outcomes: changes in PAV, TAV, FCT, lipid arc, or the proportion of patients with PAV regression. Exclusion criteria were: (i) studies not in English, (ii) animal or preclinical studies, (iii) conference abstracts, editorials, comments, or reviews, (iv) studies without a comparator group, and (v) pediatric studies (age <18 years). No restrictions were applied regarding the sex or ethnicity of the population.

All retrieved citations were imported into Rayyan, a web-based tool designed for systematic reviews and meta-analyses, for duplicate removal. Two reviewers independently screened the titles and abstracts for relevance, followed by a full-text review of potentially eligible articles. Discrepancies were resolved through discussion, and if consensus was not reached, a senior reviewer adjudicated.

A standardized data extraction template was developed and piloted on two studies prior to full application. Data were extracted independently and in duplicate by two reviewers. Extracted information included: study characteristics (design, country, year of publication, enrollment period, imaging modality, type and dose of PCSK9i, comparator, follow-up duration, sample size), baseline patient characteristics (age, sex, cardiovascular risk factors, statin use), and imaging outcomes (PAV, TAV, FCT, lipid arc, proportion of patients with PAV regression). If results were reported at multiple time points, the longest available follow-up was used. In cases of missing or unclear data, corresponding authors were contacted for clarification.

### 2.2 Risk of Bias Assessment

Two reviewers independently assessed the risk of bias at the study level and within prespecified domains. For randomized controlled trials (RCTs), we used the Cochrane RoB 2 tool, and for non-randomized comparative studies, we used the ROBINS-I tool. No studies were excluded from the quantitative synthesis on the basis of risk of bias assessment.

### 2.3 Statistical Analysis

All quantitative syntheses were conducted using the *meta*, *metafor*, and *dmetar* packages in R Studio (version 1.4.1106, RStudio, PBC, Boston, MA, USA). For continuous outcomes (PAV, TAV, lipid arc, FCT), mean differences (MDs) with 95% confidence intervals (CIs) were calculated. For dichotomous outcomes (proportion of patients with PAV regression), risk ratios (RRs) with 95% CIs were estimated. Pooled effect estimates were derived using random-effects models to account for between-study variability (restricted maximum likelihood (REML) with Hartung–Knapp).

Between-study heterogeneity was assessed using the I^2^ statistic, categorized as low (<25%), moderate (25–75%), or high (>75%). Sensitivity analyses were performed using the leave-one-out method to evaluate the robustness of the results. Potential publication bias was assessed visually through funnel plots and statistically with Egger’s regression test.

We also performed random-effects meta-regressions to explore whether between-study characteristics explained variability in the impact of PCSK9i use on the outcomes of interest. Prespecified moderators were: follow-up duration, study design (RCT vs. non-RCT), dosing regimen (monthly vs. biweekly), and type of PCSK9i (evolocumab vs. alirocumab).

## 3. Results

### 3.1 Study Selection

The search identified 142 records across databases. After de-duplication and removal of clearly irrelevant items at the identification stage, 61 unique citations proceeded to title/abstract screening. Of these, 42 were excluded for reasons such as non-original articles, absence of intracoronary imaging, ineligible interventions or comparators, or wrong population/outcomes, leaving 19 articles for full-text review. Following full-text assessment, 9 studies met the inclusion criteria and were entered into data extraction and quantitative synthesis. The remaining 10 articles represented substudies of already included studies. The PRISMA flow diagram summarizing this process is presented in **Supplementary Fig. 1**.

### 3.2 Systematic Review

The characteristics of the included studies are presented in Table 1 (Ref. [9, 10, 11, 12, 13, 14, 15, 16, 17]) and **Supplementary Table 1**. Seven of them were randomized trials (three double-blind, placebo-controlled—GLAGOV [9], HUYGENS [10], and PACMAN-AMI [11]; four open-label randomized—ODYSSEY J-IVUS [12], ALTAIR [13], Gao *et al*. [14], and Adage-Joto [15]) and two nonrandomized studies [16, 17]. Trials were conducted across multiple regions—GLAGOV at 197 sites worldwide [9], PACMAN-AMI at nine European centers [11], HUYGENS across international sites [10], and the remaining studies predominantly in Japan and East Asia.

**Table 1.** **Characteristics of the included studies**.

Study	Year	Design	Country	PCSK9i (Dose)	Population	IVI method	Follow-up (w)	Risk of bias summary
GLAGOV [9]	2016	RCT	Multiple	Evolocumab (420 mg/month)	CCS	IVUS	76	Low
ODYSSEY J-IVUS [12]	2019	RCT	Japan	Alirocumab (up to 150 mg/2 weeks)	ACS	IVUS	36	Some Concerns
Yano *et al*. [16]	2020	Non-RCT	Japan	Evolocumab (140 mg/2 weeks)	ACS	OCT	12	Serious
ALTAIR [13]	2020	RCT	Japan	Alirocumab (75 mg/2 weeks)	CCS	OCT	36	Some Concerns
Gao *et al*. [14]	2021	RCT	China	Alirocumab (75 mg/2 weeks)	Mixed	OCT	36	Some Concerns
Ota *et al*. [17]	2022	Non-RCT	Japan	Any	Mixed	IVUS	45	Serious
HUYGENS [10]	2022	RCT	Multiple	Evolocumab (420 mg/month)	ACS	OCT	52	Low
PACMAN-AMI [11]	2022	RCT	Europe	Alirocumab (150 mg/2 weeks)	ACS	IVUS	52	Low
						OCT		
Adage-Joto [15]	2024	RCT	Japan	Evolocumab (140 mg/2 weeks or 420 mg/month)	ACS	OCT	36	Some Concerns

RCT, randomized controlled trial; CCS, chronic coronary syndrome; ACS, acute coronary syndrome; IVI, intravascular imaging; IVUS, intravascular ultrasound; OCT, optical coherence tomography.

Interventions compared a PCSK9i added to background lipid-lowering therapy versus placebo or standard care. Evolocumab (typically 420 mg monthly or 140 mg every 2 weeks) and alirocumab (75–150 mg every 2 weeks) were the active agents; high-intensity statins were mandated in PACMAN-AMI [11], while several open-label studies permitted usual care (with ezetimibe allowed in some) or explicitly restricted add-on therapies [13]. Imaging assessments were performed at blinded core laboratories in the pivotal trials.

Follow-up intervals ranged from 12 to 76 weeks. Across studies, patient populations spanned stable CAD (GLAGOV) and post-ACS settings (HUYGENS, PACMAN-AMI, ODYSSEY J-IVUS, Yano, Adage-Joto), with ALTAIR specifically enrolling OCT-defined thin-cap fibroatheroma after PCI.

Across randomized evidence, overall risk of bias was low in the double-blind, placebo-controlled trials—GLAGOV, HUYGENS, and PACMAN-AMI—owing to robust randomization/masking and centralized, blinded core-lab imaging, whereas open-label randomized studies were judged as “some concerns” primarily for deviations from intended interventions and limited concealment despite blinded outcome assessment (**Supplementary Table 2**). Nonrandomized studies showed a serious risk of bias dominated by confounding and selection (treatment-by-indication and self-selection), even though imaging analysis was blinded.

### 3.3 Meta-Analysis

#### 3.3.1 IVUS Parameters

**Fig. 1.**
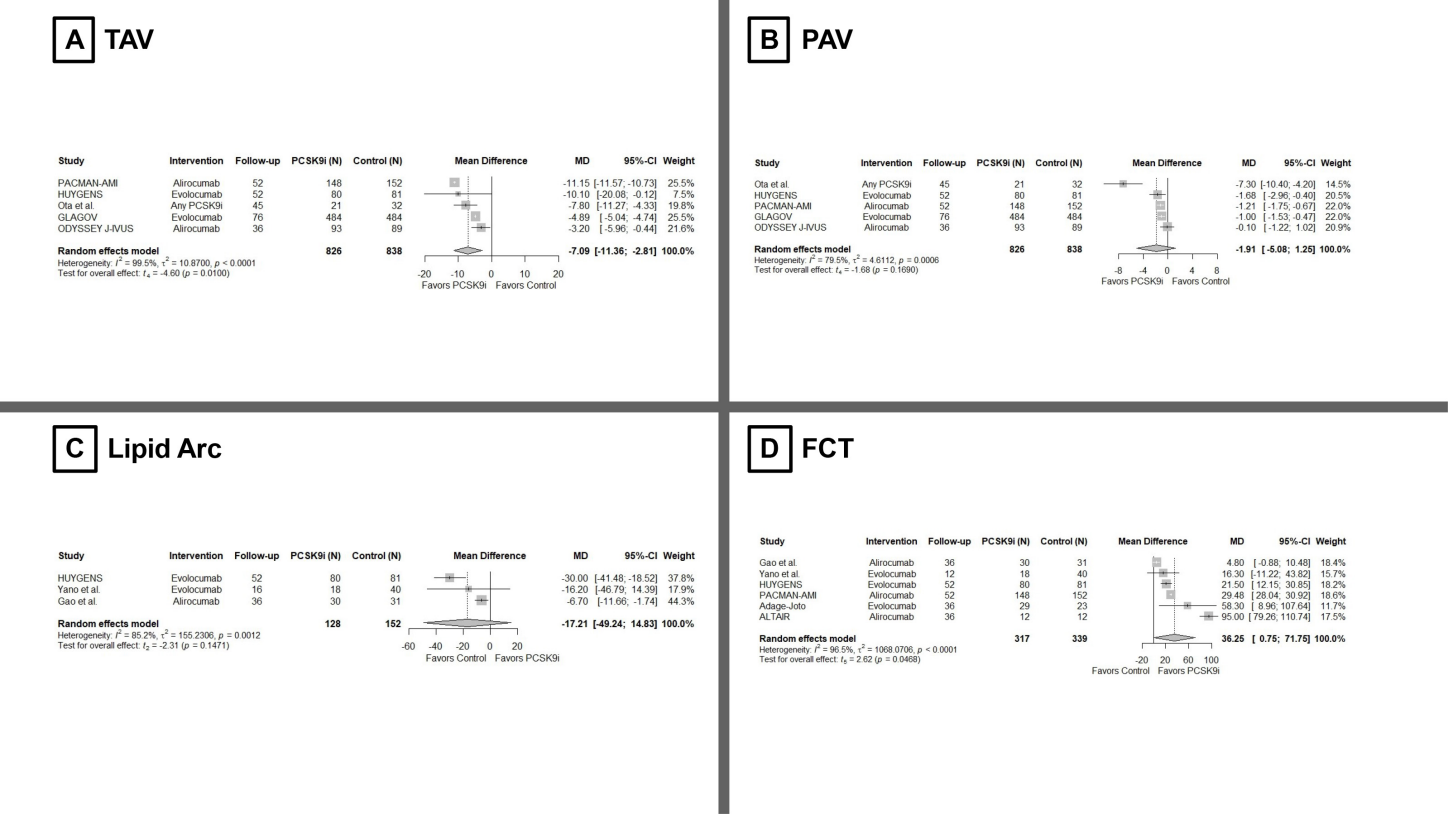
**Meta-analysis on the effect of PCSK9is on (A) TAV, (B) PAV, (C) Lipid Arc, and (D) FCT**. PCSK9is, Proprotein convertase subtilisin/kexin type 9 inhibitors; TAV, total atheroma volume; PAV, percent atheroma volume; FCT, fibrous cap thickness.

Five studies contributed to the TAV change. PCSK9is produced a greater reduction in TAV versus controls (Fig. 1A, MD –7.09 mm^3^, 95% CI –11.36 to –2.81; *p* = 0.01). Between-study heterogeneity was considerable (I^2^ = 100%; *p* < 0.001). PCSK9i therapy yielded a larger reduction in PAV compared with controls, albeit not statistically significant (Fig. 1B, MD –1.91%, 95% CI –5.08 to 1.25; *p* = 0.17). Heterogeneity was high (I^2^ = 80%; *p* < 0.001). Pooling GLAGOV, HUYGENS, and PACMAN-AMI, the proportion of patients with PAV regression was higher with PCSK9is (RR 1.30, 95% CI 1.19 to 1.42; *p* < 0.001), with very low heterogeneity (I^2^ = 0%; τ^2^ ≈ 0; *p* = 0.38).

#### 3.3.2 OCT Parameters

Across three studies, PCSK9 inhibition was associated with a trend toward a smaller lipid arc versus comparators (Fig. 1C, MD –17.64°, 95% CI –49.73 to 14.44; *p* = 0.14), with substantial heterogeneity (I^2^ = 79%; *p* for heterogeneity = 0.009). The direction of effect was consistent across all studies, but the pooled estimate did not reach statistical significance. Across six studies, PCSK9 inhibition led to a greater increase in minimum FCT (Fig. 1D, MD 36.25 µm, 95% CI 0.75 to 71.75; *p* = 0.047), consistent with a plaque-stabilizing effect, although this association was borderline statistically significant. Heterogeneity was high (I^2^ = 97%; *p* < 0.001), consistent with variability in OCT acquisition/analysis windows and timing of repeat imaging.

#### 3.3.3 Publication Bias

Visual inspection of funnel plots (**Supplementary Figs. 2–5**) and Egger’s test for TAV, PAV, lipid arc, and FCT did not reveal any clear evidence of small-study effects; however, the power of funnel plots and Egger’s test is limited given the small number of studies.

As a sensitivity analysis, we applied the trim-and-fill method for all endpoints (**Supplementary Table 3**). For PAV and TAV, imputing 1 and 2 potentially missing studies, respectively, yielded adjusted pooled effects that remained similar in magnitude and direction to the primary estimates, with TAV still statistically significant. In contrast, for FCT, the imputed analysis attenuated the effect, and the association was no longer statistically significant, while lipid arc remained non-significant. Overall, these findings suggest that potential publication bias is unlikely to fully explain our main results, but the small number of studies—particularly for FCT and lipid arc—warrants cautious interpretation.

#### 3.3.4 Sensitivity Analysis

The results of the meta-analyses for PAV, TAV, lipid arc, and FCT remained unaffected even after exclusion of any single study using the Leave-One-Out method (**Supplementary Figs. 6–9**).

To explore potential sources of between-study heterogeneity, we performed random-effects meta-regression including follow-up duration, study design (randomized controlled trial vs. non-randomized), PCSK9i regimen (monthly vs. other), and, where applicable, type of PCSK9i (evolocumab vs. alirocumab) as covariates (**Supplementary Table 4**). For PAV, study design emerged as a significant moderator of the treatment effect (β = 6.25, 95% CI 0.36 to 12.15; *p* = 0.04), accounting for essentially all of the between-study variance (R^2^ = 100%, residual I^2^ = 0%). In contrast, neither follow-up duration (β = 0.03; *p* = 0.81) nor dosing regimen (β = –0.39; *p* = 0.59) was associated with changes in PAV, and both left residual heterogeneity largely unchanged. Follow-up duration, study design, and regimen all showed non-significant associations with TAV (all *p* ≥ 0.86) and FCT (all *p* ≥ 0.59), and residual heterogeneity remained very high (I^2^ > 90% for all models), indicating that the substantial between-study variability for these outcomes is largely driven by unmeasured factors. For lipid arc, meta-regression suggested that treatment type and dosing regimen might contribute to heterogeneity. Models including evolocumab use or monthly dosing yielded R^2^ values of 100% with residual I^2^ = 0%. However, the corresponding regression coefficients were not statistically significant (β = –21.6; *p* = 0.17 for evolocumab; β = –22.5; *p* = 0.12 for monthly regimen), and confidence intervals were wide, consistent with limited power.

## 4. Discussion

This meta-analysis suggests that adding a PCSK9i to background lipid-lowering therapy may favorably influence coronary atherosclerotic plaque as assessed by intravascular imaging. We observed a significant reduction in IVUS-derived TAV and a higher proportion of patients achieving PAV regression with PCSK9is, while the pooled change in PAV itself did not reach statistical significance. On OCT, PCSK9i use was associated with a modest increase in minimum FCT and a non-significant trend toward a smaller lipid arc. Among these endpoints, the proportion of patients with PAV regression showed the most consistent effect with negligible heterogeneity, whereas volumetric and compositional change metrics were characterized by substantial between-study variability. Taken together, these patterns are compatible with a shift toward a more stable plaque phenotype but should be interpreted as mechanistic and hypothesis-generating rather than definitive.

For quantitative IVUS and OCT metrics, heterogeneity was high. Several factors are likely to contribute. First, study populations ranged from stable coronary disease to acute coronary syndromes, with different baseline plaque biology and healing trajectories. Second, imaging protocols and target segments varied (e.g., non-infarct vs culprit-adjacent vessels; segment length; pullback standardization), and OCT analyses used different cap-sampling strategies. Third, exposure intensity differed by agent and timing; some studies mandated high-intensity statins and early PCSK9i initiation after PCI, whereas others allowed usual care or included short “induction” courses. Finally, follow-up intervals spanned weeks to over a year, mixing early compositional changes—such as rapid cap thickening—with slower volumetric changes captured by IVUS. Despite these sources of heterogeneity, the direction of effect was consistently in favor of PCSK9is; however, the IVUS-derived volumetric findings, in particular, should be interpreted as exploratory and hypothesis-generating.

The biological plausibility of these findings is strong. PCSK9is drive profound LDL-C lowering and modestly reduce Lp(a) [18], which together are expected to lessen lipid influx, dampen inflammation, and promote cap collagen deposition. The OCT signal—thicker caps with reduced lipid arc—aligns with a stabilization phenotype, while the IVUS signal—lower PAV/TAV and more patients achieving regression—suggests parallel effects on overall plaque burden. The convergence of modality-specific markers supports a mechanistic pathway by which intensive atherogenic-lipoprotein reduction translates into both compositional and volumetric benefits [19].

### Limitations

Several limitations should be acknowledged. First, the review was not pre-registered, which may increase the risk of selective reporting or analytic flexibility, despite our attempt to follow a predefined strategy. Second, our review was limited to English-language articles, which may have introduced selection bias and underrepresented evidence from non-English-speaking regions, thus affecting the generalizability of our findings. Third, the number of available imaging studies was small for several endpoints, and statistical heterogeneity was very high for key outcomes such as IVUS-derived PAV and TAV, so the pooled estimates should be interpreted with caution and viewed as exploratory rather than definitive. In line with this, sensitivity analyses using trim-and-fill suggested that the association between PCSK9i and increased FCT may not be robust to potential publication bias, further underscoring the exploratory nature of this finding. Fourth, there was substantial variability in intravascular imaging modalities (IVUS vs. OCT), lesion or segment selection, and analysis windows across trials, all of which may introduce methodological heterogeneity and affect comparability of absolute effect sizes. Fifth, our dataset included open-label and non-randomized studies in addition to randomized controlled trials, which raises the possibility of selection and treatment biases that cannot be fully accounted for by random-effects modeling. Moreover, follow-up duration differed markedly between studies, and although meta-regression did not identify a statistically significant impact of follow-up time on treatment effects, we cannot exclude time-dependent differences in plaque responses. Furthermore, most included participants were high-risk, or ACS patients treated predominantly with evolocumab or alirocumab on top of intensive statin therapy; as a result, the generalizability of our findings to other PCSK9-targeting agents, such as inclisiran, to lower-risk population or to less intensive background lipid-lowering regimens is limited and requires confirmation in future dedicated imaging studies. Additionally, although PCSK9is lower Lp(a) and Lp(a) reduction has been proposed as a potential contributor to plaque stabilization, Lp(a) concentrations and their longitudinal changes were infrequently and inconsistently reported across the included trials. We were therefore unable to examine the relationship between Lp(a) trajectories and intravascular imaging outcomes. Finally, our synthesis was limited to intravascular imaging outcomes. The included studies were small mechanistic trials with relatively short follow-up and infrequent, heterogeneously defined clinical events, which precluded any robust pooled analysis of MACE or formal evaluation of whether imaging changes translate into improved prognosis. Accordingly, our findings should be viewed as mechanistic and hypothesis-generating rather than as direct evidence of clinical benefit, and they need to be interpreted alongside the results of large outcome trials of PCSK9is.

## 5. Conclusions

Across a limited number of small and heterogeneous imaging studies, PCSK9 inhibition added to standard lipid-lowering therapy appears to be associated with modest reductions in IVUS-defined TAV, a higher proportion of patients achieving PAV regression, and small increases in OCT-derived minimal FCT. These findings should be viewed as exploratory and hypothesis-generating, providing suggestive mechanistic support that intensified LDL-lowering with PCSK9i may favorably influence coronary plaque burden and phenotype in high-risk patients, but require confirmation in larger, dedicated imaging trials with standardized imaging protocols.

## Availability of Data and Materials

The datasets supporting this article are available upon reasonable request from the corresponding author.

## Supplementary Material

Supplementary material associated with this article can be found, in the online version, at https://doi.org/10.31083/RCM46547.
