# Supplementary material for: Proprotein Convertase Subtilisin/Kexin Type 9 and Atherosclerotic Plaque Progression: A Systematic Review and Meta-Analysis of Intravascular Imaging Studies
Source: Rev Cardiovasc Med. 2026 Mar 10;27(3):46547. doi: 10.31083/RCM46547 (PMC13036525; doi:10.31083/RCM46547)
Supplement: Supplementary file 1 [file 2153-8174-27-3-46547-s1.zip › Supplementary Material.docx]

**Supplementary Material**

Contents

[**Supplementary Table 1**. Baseline characteristics of the populations in the respective studies. 2](#_Toc221881680)

[**Supplementary Table 2**. Risk of Bias assessment of the included studies. 3](#_Toc221881681)

[**Supplementary Table 3.** Egger’s test and trim-and-fill analysis. 4](#_Toc221881682)

[**Supplementary Table 4.** Meta-regression analysis concerning the effect of extracted variables on the outcomes of interest. 5](#_Toc221881683)

[**Supplementary Figure 1.** PRISMA flowchart for the study selection process. 6](#_Toc221881684)

[**Supplementary Figure 2.** Funnel plot of studies evaluating the impact of PCSK9 inhibitors on TAV compared to placebo. 7](#_Toc221881685)

[**Supplementary Figure 3.** Funnel plot of studies evaluating the impact of PCSK9 inhibitors on PAV compared to placebo. 8](#_Toc221881686)

[**Supplementary Figure 4.** Funnel plot of studies evaluating the impact of PCSK9 inhibitors on lipid arc compared to placebo. 9](#_Toc221881687)

[**Supplementary Figure 5.** Funnel plot of studies evaluating the impact of PCSK9 inhibitors on FCT compared to placebo. 10](#_Toc221881688)

[**Supplementary Figure 6.** Leave-one-out sensitivity analysis on the meta-analysis evaluating the impact of PCSK9 inhibitors on TAV compared to placebo. 11](#_Toc221881689)

[**Supplementary Figure 7.** Leave-one-out sensitivity analysis on the meta-analysis evaluating the impact of PCSK9 inhibitors on PAV compared to placebo. 12](#_Toc221881690)

[**Supplementary Figure 8.** Leave-one-out sensitivity analysis on the meta-analysis evaluating the impact of PCSK9 inhibitors on lipid arc compared to placebo. 13](#_Toc221881691)

[**Supplementary Figure 9.** Leave-one-out sensitivity analysis on the meta-analysis evaluating the impact of PCSK9 inhibitors on FCT compared to placebo. 14](#_Toc221881692)

# **Supplementary Table 1**. Baseline characteristics of the populations in the respective studies.

| **Study** | **Mean age (I)** | **Mean age (C)** | **Male sex (I)** | **Male sex (C)** | **HTN (I)** | **HTN (C)** | **DM (I)** | **DM (C)** | **Statin (I)** | **Statin (C)** |
| --- | --- | --- | --- | --- | --- | --- | --- | --- | --- | --- |
| GLAGOV | 59.8 | 59.8 | 72.1 | 72.3 | 82.2 | 83.7 | 20.2 | 21.5 | 98.8 | 98.3 |
| HUYGENS | 60.9 | 60.2 | 75 | 67.9 | 56.3 | 40.7 | 16.3 | 17.3 | 93.8 | 96.3 |
| PACMAN‑AMI | 58.4 | 58.6 | 83.8 | 78.3 | 40.5 | 46.1 | 8.1 | 12.5 | 100 | 100 |
| ODYSSEY J‑IVUS | 61.8 | 60.5 | 79.6 | 80.9 | 68.8 | 70.8 | 29 | 34.8 | 100 | 100 |
| Gao *et al.* | 61.3 | 61.3 | 66.7 | 74.2 | 56.7 | 61.3 | 23.3 | 25.8 | 100 | NR |
| ALTAIR | NR | NR | NR | NR | NR | NR | NR | NR | 100 | 100 |
| Adage‑Joto | 59.6 | 60 | 75.8 | 69.5 | 55.8 | 60.1 | 34.4 | 34.7 | 100 | 100 |
| Ota *et al.* | 63.8 | 69.5 | 76.2 | 62.5 | 81 | 93.8 | 38.1 | 15.6 | 100 | 100 |
| Yano *et al.* | 64.6 | 65.2 | 77.8 | 77.5 | 72.2 | 70 | 44.4 | 37.5 | 100 | 100 |
| I: intervention, C: control, HTN: hypertension, DM: diabetes mellitus, NR: not reported | | | | | | | | | | |

# **Supplementary Table 2**. Risk of Bias assessment of the included studies.

| **Study (Year)** | **Assessment tool** | **Key domains with concern** | **Overall RoB** |
| --- | --- | --- | --- |
| GLAGOV (2016) | RoB 2 | None noted (randomization, deviations, missing, measurement, reporting all low) | Low |
| HUYGENS (2022) | RoB 2 | None noted; prespecified SAP; blinded imaging analysts | Low |
| PACMAN‑AMI (2022) | RoB 2 | None noted; robust blinding; core‑lab imaging; prespecified endpoints | Low |
| ODYSSEY J‑IVUS (2019) | RoB 2 | Some concerns for deviations from intended interventions (open‑label); others low | Some concerns |
| Gao et al. (2021) | RoB 2 | Randomization process not fully described; open‑label → some concerns for deviations; imaging analysis blinded | Some concerns |
| ALTAIR (2020) | RoB 2 | Small sample; allocation concealment not clear; open‑label; blinded core‑lab imaging | Some concerns |
| Adage‑Joto (2024) | RoB 2 | Open‑label; early discontinuation and SoC changes may introduce deviations; core‑lab imaging masked | Some concerns |
| Ota et al. (2022) | ROBINS‑I | Serious risk due to confounding/selection; objective blinded imaging lowers measurement bias | Serious |
| Yano et al. (2020) | ROBINS‑I | Serious risk from confounding and selection; objective imaging lowers measurement bias | Serious |

# **Supplementary Table 3.** Egger’s test and trim-and-fill analysis.

|  | **Egger’s test (p-value)** | **Added studies** | **MD** | **95% CI** | **P** | **I^2^** |
| --- | --- | --- | --- | --- | --- | --- |
| **TAV** | 0.57 | 2 | -5.06 | -9.51, -0.62 | 0.03 | 99.7% |
| **PAV** | 0.29 | 1 | -1.01 | -4.96, 2.93 | 0.54 | 85.8% |
| **PAV regression** | 0.87 | 0 | 1.30 | 1.19, 1.42 | <0.001 | 0% |
| **Lipid arc** | 0.86 | 2 | -6.7 | -29.3, 16.5 | 0.47 | 87.7% |
| **FCT** | 0.96 | 1 | 25.4 | -13.7, 64.6 | 0.16 | 97.2% |

# **Supplementary Table 4.** Meta-regression analysis concerning the effect of extracted variables on the outcomes of interest.

| **Covariate** | **β** | **P value^†^** | **CI** | **R^2 ‡^** | **I^2 ˠ^** |
| --- | --- | --- | --- | --- | --- |
| **PAV** |  |  |  |  |  |
| Follow-up duration | 0.0265 | 0.81 | -0.29, 0.35 | 0% | 96.5% |
| Study design (RCT) | 6.2517 | 0.04 | 0.36, 12.15 | 100% | 0% |
| Regimen (Monthly) | -0.3938 | 0.59 | -3.06, 2.28 | 0% | 49.79 |
| **TAV** |  |  |  |  |  |
| Follow-up duration | 0.0022 | 0.99 | -0.39, 0.40 | 0 | 93.65% |
| Study design (RCT) | 0.8628 | 0.86 | -13.08, 14.80 | 0 | 99.5% |
| Regimen (Monthly) | 0.8275 | 0.88 | -19.98, 21.64 | 0 | 92% |
| **FCT** |  |  |  |  |  |
| Follow-up duration | 0.076 | 0.95 | -3.11, 3.26 | 0 | 98% |
| Study design (RCT) | 23.8680 | 0.59 | -88.67, 136.41 | 0 | 99% |
| Intervention (Evolocumab) | -12.8492 | 0.69 | -96.89, 71.19 | 0% | 98.83% |
| **LA** |  |  |  |  |  |
| Follow-up duration | -0.4083 | 0.62 | -8.12, 7.31 | 0 | 76.55% |
| Study design (RCT) | -1.9038 | 0.95 | -318.83, 315.02 | 0 | 89.5% |
| Intervention (Evolocumab) | -21.5969 | 0.17 | -98.09, 54.90 | 100% | 0% |
| Regimen (Monthly) | -22.4862 | 0.12 | -77.41, 32.44 | 100% | 0% |

† P-value from a Wald test for the effect of the covariate in the model

‡ R2, the relative reduction in the between-study variance

ˠ I2, the percentage of the residual variation that is attributable to between-study heterogeneity


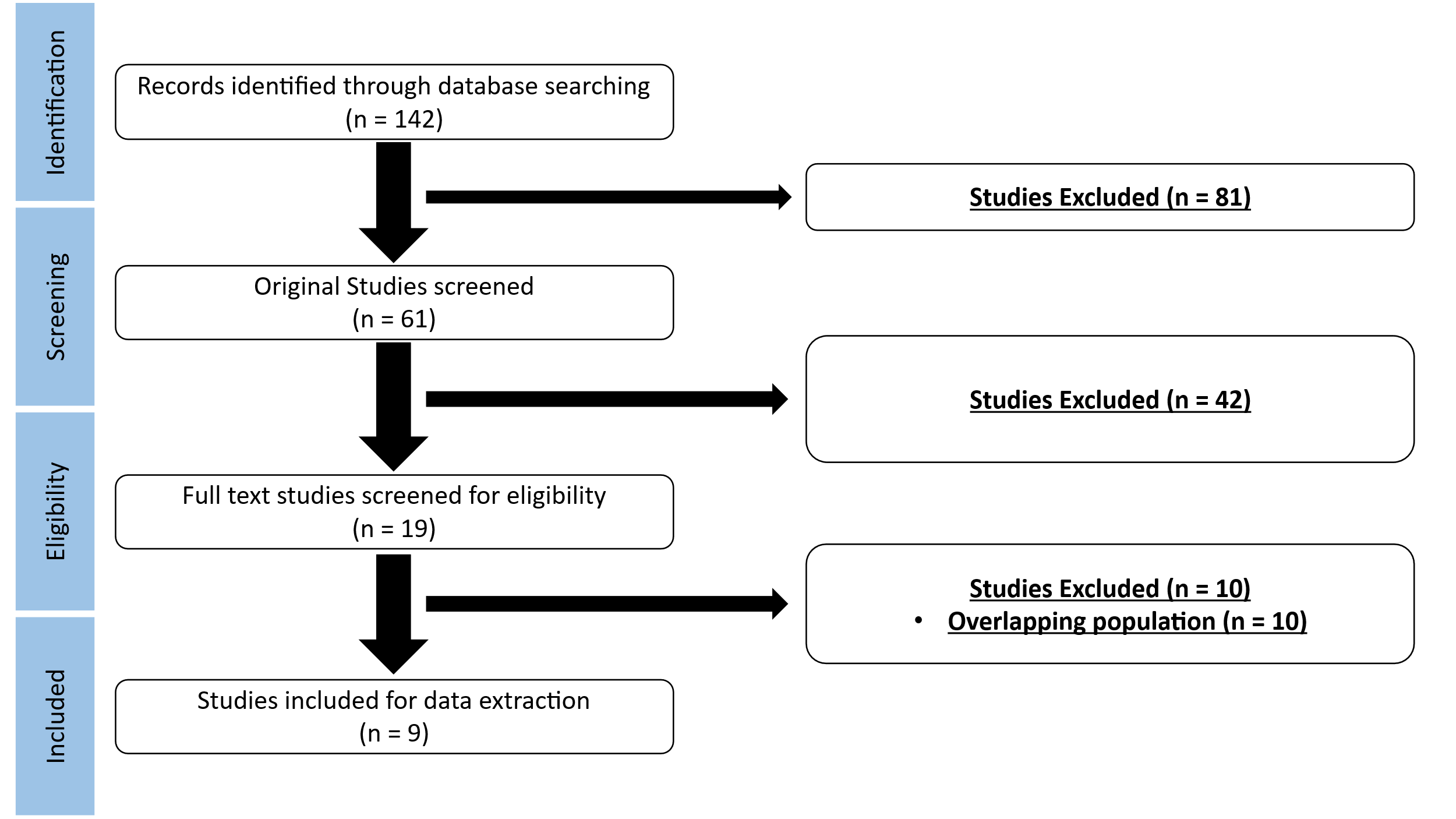


# **Supplementary Figure 1.** PRISMA flowchart for the study selection process.


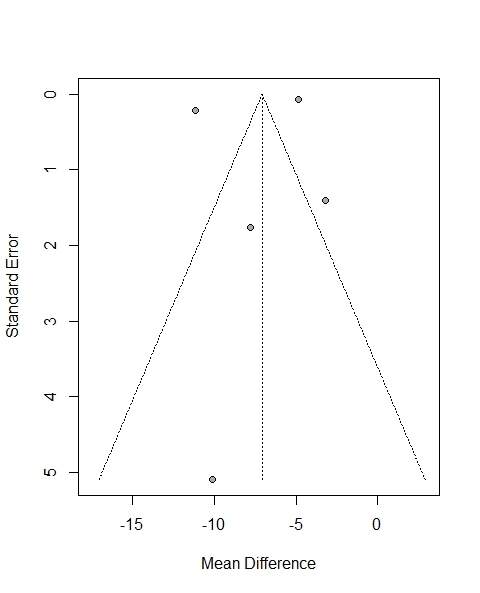


# **Supplementary Figure 2.** Funnel plot of studies evaluating the impact of PCSK9 inhibitors on TAV compared to placebo.


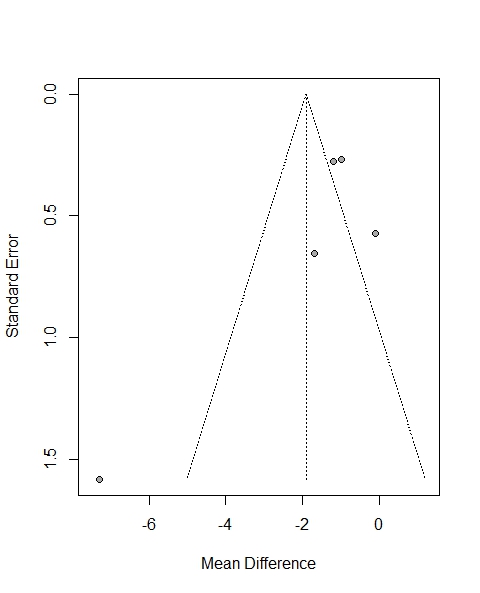


# **Supplementary Figure 3.** Funnel plot of studies evaluating the impact of PCSK9 inhibitors on PAV compared to placebo.


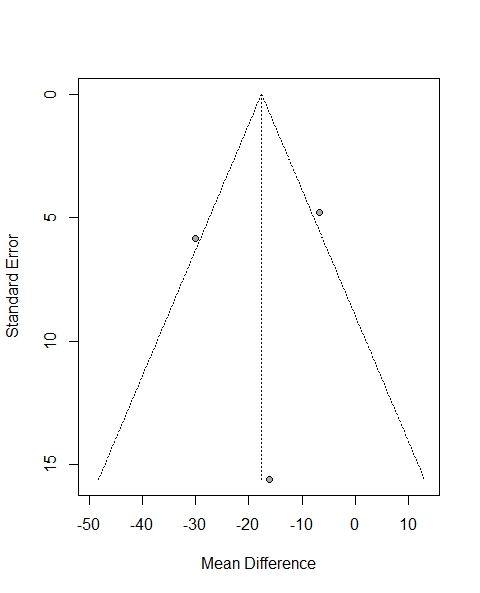


# **Supplementary Figure 4.** Funnel plot of studies evaluating the impact of PCSK9 inhibitors on lipid arc compared to placebo.


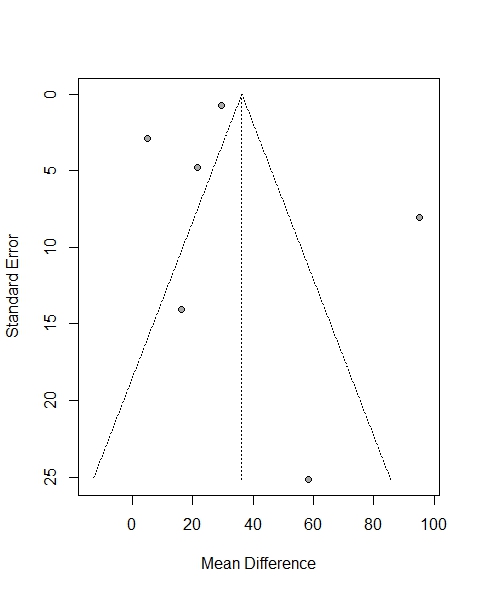


# **Supplementary Figure 5.** Funnel plot of studies evaluating the impact of PCSK9 inhibitors on FCT compared to placebo.


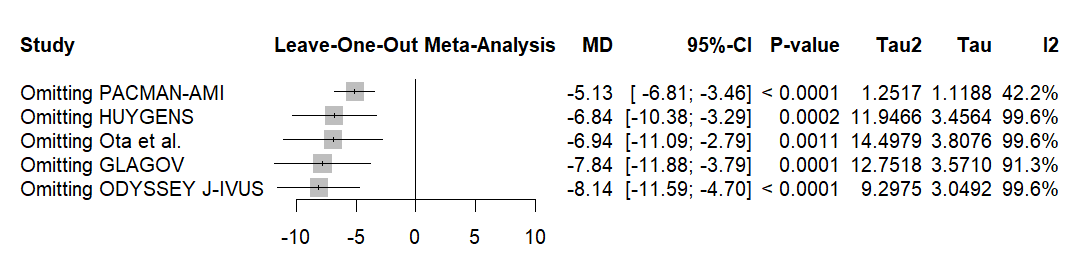


# **Supplementary Figure 6.** Leave-one-out sensitivity analysis on the meta-analysis evaluating the impact of PCSK9 inhibitors on TAV compared to placebo.


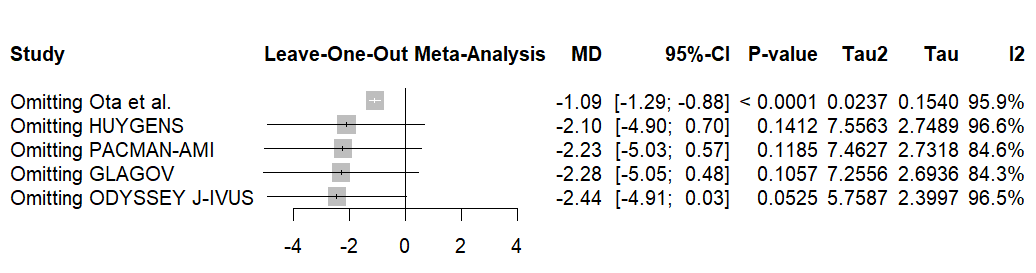


# **Supplementary Figure 7.** Leave-one-out sensitivity analysis on the meta-analysis evaluating the impact of PCSK9 inhibitors on PAV compared to placebo.


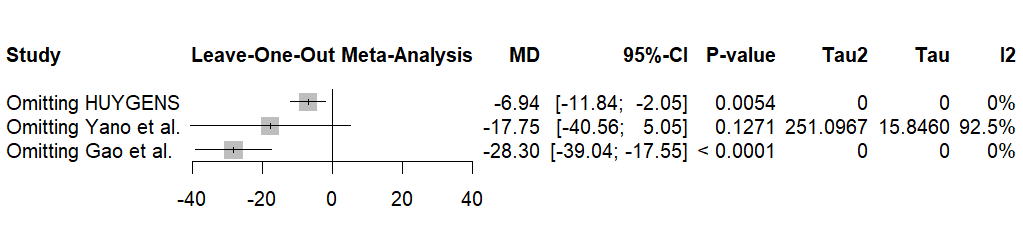


# **Supplementary Figure 8.** Leave-one-out sensitivity analysis on the meta-analysis evaluating the impact of PCSK9 inhibitors on lipid arc compared to placebo.


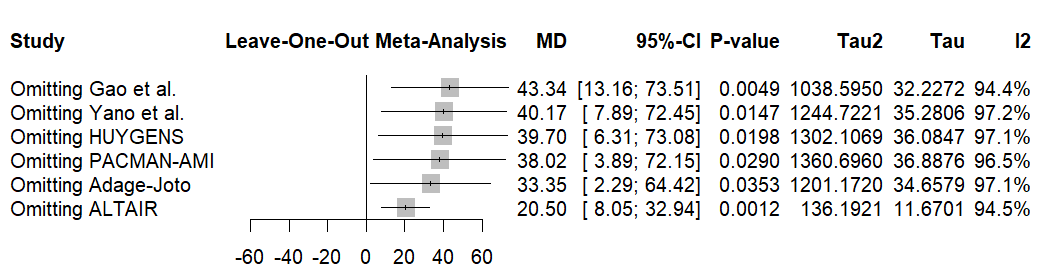


# **Supplementary Figure 9.** Leave-one-out sensitivity analysis on the meta-analysis evaluating the impact of PCSK9 inhibitors on FCT compared to placebo.
